# Association of GAB1 gene with asthma susceptibility and the efficacy of inhaled corticosteroids in children

**DOI:** 10.1186/s12890-023-02790-0

**Published:** 2023-12-06

**Authors:** Yuxuan Zhang, Jun Liu, Yanjie Zhi, Xuan You, Bing Wei

**Affiliations:** 1 Department of neonatology, General Hospital of Northern Theater Command, Shenyang, 110016 Liaoning People’s Republic of China; 2 Post-graduate College, Jinzhou Medical University, Jinzhou, 121000 Liaoning People’s Republic of China

**Keywords:** Childhood asthma, Environment, *GAB1*, Inhaled corticosteroids, Interaction, Variant

## Abstract

Asthma is a polygenic disease that may onset during childhood. Inhaled corticosteroids (ICS) are the main therapy in asthma, although their efficacy varies among individuals. Nuclear factor κB (NF-κB) is an important target of ICS treatment of asthma. Recent research has reported that GRB2 associated binding protein 1 (*GAB1*) gene may participate in the pathogenesis of asthma by regulating the NF-κB pathway. Therefore, we used the technique of an improved multiplex ligation detection reaction to sequence *GAB1* gene and investigated the involvement of Single-nucleotide variants (SNVs) in *GAB1* gene in asthma and ICS efficacy in asthmatic children. We found no differences between asthma cases and controls in allele or genotype frequencies of *GAB1*. Haplotype analysis showed an increased tendency for AGGAGC frequency in asthma patients compared with controls (OR = 2.69, *p* = 0.018). The percentage of EOS and genotype distribution of rs1397527 were associated (*p* = 0.007). The EOS percentage was higher in GT genotype when compared to the GG genotype (5.50 vs 3.00, Bonferroni adjusted *p* = 0.005). After 12-weeks ICS treatment, *GAB1* rs1397527 TT and GT genotype carriers had a smaller change in forced expiratory volume in 1 second/forced vital capacity (FEV1/FVC) than GG carriers (*p* = 0.009), and rs3805236 GG and AG genotype carriers also had a smaller change in FEV1/FVC than AA carriers (*p* = 0.025). For ICS response, the frequency of GG genotype of rs1397527 was significantly higher in good responders (*p* = 0.038). The generalized multifactor dimensionality reduction (GMDR) analysis showed a best significant four-order model (rs1397527, allergen exposure, environmental tobacco smoke exposure, and pet exposure) involving gene-environment interactions (*p* = 0.001). In summary, we found that *GAB1* SNVs were not associated with asthma susceptibility. Haplotype AGGAGC was a risk factor for asthma. *GAB1* variants were associated with eosinophils and ICS response in asthmatics. Furthermore, gene-environment interaction was observed.

**Supplementary Information:**

The online version contains supplementary material available at 10.1186/s12890-023-02790-0.

## Introduction

Asthma is the most common chronic respiratory disease, characterized by chronic airway inflammation and hyper-responsiveness, implicating various inflammatory cells and cytokines [1]. Asthma symptoms typically first occur in childhood, mainly represented as wheeze, shortness of breath, chest tightness and cough that vary over time and intensity. The severity of childhood asthma strongly affects lung function and prognosis in adult life [2]. Recent years have witnessed a growing number of patients with asthma. According to the Global Burden of Disease Collaboration in 2019, as many as 262 million people worldwide suffer from asthma [3], which has become a heavy burden on families and society. It is generally accepted that asthma is a polygenic disease [4]. Studies on twins and families with asthma cases have revealed that genetic variation also plays a crucial role in the pathogenesis of childhood asthma. According to a meta-analysis of 71 twin studies, the heritability of asthma can be as high as 0.54 [5]. Furthermore, environmental factors can influence the frequency and severity of asthma attacks via epigenetic mechanisms [6]. A study has found that environmental exposures (e.g., allergens, air pollutants, specific dietary components) can induce changes in DNA methylation and histone post-translational modifications (e.g., acetylation, or phosphorylation) that may modify regulatory mechanisms that affect asthma-related gene expression [7].

Inhaled corticosteroids (ICS), the first-line drug recommended by the Global Initiative for Asthma (GINA), can alleviate bronchial mucosal congestion, edema, and inflammatory exudation. They are currently the most widely used and effective medication for asthma and play a crucial role in improving children’s pulmonary function and preventing disease progression. They bind to and interact with glucocorticoid receptors, regulate multiple signaling pathways and affect the expression and transcription of numerous genes involved in the inflammatory process, including lowering proinflammatory gene expression and increasing anti-inflammatory gene expression. However, clinical practice and research have discovered that the efficacy of ICS for asthma patients varies from person to person [8], and this heterogeneity has a genetic basis [9], which may be related to variants in the sequence of effector genes. Pharmacogenomics has discovered a link between multiple gene SNVs and the efficacy of ICS [10]. Identifying variants that contribute to asthma treatment response would allow genetic information to be used to pick the most appropriate asthma therapy for individuals [11].

Among the numerous inflammatory factors and pathways involved in the pathogenesis of asthma, nuclear factor kappa B (NF-κB) is considered as one of the essential transcription factors [12] because of its role in regulating the transcription of cytokines, adhesion molecules, and chemokines in allergic inflammation. In addition, it is one of the main targets of treatment with ICS in asthma [13]. The upregulation of its expression is related to the poor efficacy of ICS and asthma exacerbating or worsening [14]. Recent studies have confirmed that the expression of the GRB2 associated binding protein 1 (*GAB1*) gene may be related to the pathogenesis of asthma. *GAB1* is expressed in alveolar type II cells (AT-IIs) and can promote the production of inflammatory cytokines in macrophages by the NF-κB pathway activation [15]. Only one study reported that the LD block region including rs1397527 contained the *GAB1* gene and was locus susceptible to asthma in adults [16]. In consideration of the limited number of studies on relationship between *GAB1* gene variants and asthma, the current study aimed to illustrate the association of genetic variation in *GAB1* gene with the risk for asthma and the ICS response in asthmatic children, and to assess the modulatory effect of interactions in SNV-SNV and gene-environment between these variants on disease risk.

## Methods

### Patients

Between October 2021 to October 2022, 109 children with acute asthma attacks were continuously enrolled as the asthma group from the pediatric outpatient department of General Hospital of Northern Theater Command, with no repeated visits in the cohort. The inclusion criteria were the following: (1) met the diagnostic requirements outlined in the “Guideline for the diagnosis and optimal management of asthma in children” [17], which closely followed the GINA (2015); (2) had no history of systemic or local hormone and ICS medication within 4 weeks prior to enrollment, and no bronchodilator was used within 24 hours. Exclusion criteria: (1) patients with congenital lung malformations, airway obstruction or extraluminal oppression, active tuberculosis, bronchiectasis, congenital heart disease, or liver and kidney dysfunction; (2) children who had a history of allergy or intolerance to ICS (budesonide, for example). In addition, 158 healthy children who attended the hospital’s pediatric outpatient clinic during the same period were chosen as the control group, all with no history of asthma diseases.

All participants’ clinical information was obtained, including biological sex, age, body mass index (BMI), feeding patterns, allergen exposure, pet exposure, environmental tobacco smoke (ETS) exposure, and residence. BMI was calculated as weight/height^2^ (kg/m^2^). The variable feeding patterns during the first 6 months of an infant’s life was categorized into breastfeeding and artificial/mixed feeding. Allergen exposure referred to the result level of an allergen test being one or greater than one, defined as allergen positive. Pet exposure referred to keeping a dog/cat/rodent indoors. ETS exposure was defined as any smoking in the child’s home. The type of residence was categorized into urban and rural. This study was performed in line with the principles of the Declaration of Helsinki. The ethics committee of the General Hospital of Northern Theater Command approved the study. All selected minors’ parents or legal guardians completed an informed consent form.

### DNA extraction and genotyping

Three ml of peripheral venous blood from each child was collected, placed in an EDTA-Na anticoagulant tube, and kept at − 80 °C. DNA was extracted from blood samples using a DNA extraction kit (Genesky Biotechnologies Inc., Shanghai, 201,315) according to the manufacturer’s instructions. First, protease K solution was added to the test tube, next blood samples was added. Then, genomic DNA was prepared from peripheral blood leukocytes according to standard procedures with the Nucleic Acid Extraction System (magnetic-bead method). PCR primers were designed by online Primer3 software and provided by Shanghai Sheng Gong Company, and used to expand gene loci; primers information is listed in Table 1. Improved multiplex ligation detection reaction (iMLDR™) was used to genotype GAB1 SNVs (rs1397527, rs17017425, rs1866756, rs3805236, rs3805246, and rs3805254) and identify variants in the gene fragment from the gene reference sequence.

**Table 1:** Primers information

SNVs	Major allele > Minor allele	Position	F-seq (5′-3′)	R-seq (5′-3′)	Length
rs1397527	G > T	Chr4:144326385	CCCTCTGAGAGACTGGGAAGTCA	TGGGACTGAAACCGTGCCAATA	207
rs17017425	C > T	Chr4:144165951	CCTGGCTCAGCTTCTTCCTTTC	GCCAATCCTCTTCCATATTCAGTTTC	304
rs1866756	G > A	Chr4:144300064	TGCAGCTGACCCTTTGTATCTCC	GTAGTCCCCAGCAACCCCTACC	298
rs3805236	A > G	Chr4:144357737	GGGCCCTTACTAGGTTTTTCTCTTGA	GGCACCGAGCCCTTCTTCTTAG	242
rs3805246	G > A	Chr4:144304108	TGACTCTGTTCCCTAATCGTGAGGA	TGGGCCAGAGTCATTACAACACA	316
rs3805254	A > G	Chr4:144285683	GCATGGGCTTGTGAAATCTCCT	GGACACCTAGCCCCACAAACAT	288

### Determination of eosinophils and immunoglobulin E (IgE) in peripheral blood

Two milliliters of venous blood were collected from children in the asthma group, and the percentage of peripheral blood eosinophils (EOS) was measured using an LH780 blood cell analyzer; IgE was determined using an enzyme immunofluorescence assay by the UniCAP100 fully automated fluorescence detection system.

### Pulmonary function test

For children aged 5 and above with asthma, we determined pulmonary function by the JAEGER Master Screen spirometry system before and after 12-weeks of ICS treatment, including the percentage of predicted forced vital capacity (FVC% pred), percentage of predicted forced expiratory volume in 1 s (FEV1% pred), the Tiffenau index (FEV1/FVC), percentage of predicted peak expiratory flow (PEF% pred), percentage of predicted forced expiratory flow at 25% of forced vital capacity (FEF25% pred), FEF50% pred, FEF75% pred, percentage of predicted maximal mid-expiratory flow (MMEF% pred). Repeated the measurement three times, and took the best one among the patient’s cooperation, pulmonary function images, and data.

### Evaluation of ICS efficacy

ICS efficacy was evaluated as the change in FEV1/FVC, calculated as FEV1/FVCtreatment – FEV1/FVCbaseline, and the percent change in FEV1 (Δ FEV1), calculated as (FEV1treatment – FEV1baseline)/ FEV1baseline × 100%, after 12-weeks of ICS treatment. Participants were categorized as ICS responders (Δ FEV1 ≥ 8%) or nonresponders (Δ FEV1 < 8%) based on an 8% FEV1 improvement threshold, which has been demonstrated to be a good predictor of asthma treatment response in children [18–20].

### Statistical analysis

Statistical analyses were conducted by using SPSS (version 25.0). The Hardy-Weinberg equilibrium (HWE) was used to estimate allele frequencies and deviations of the genotype. Using mean ± standard deviation (SD) or median and interquartile range (IQR) to describe continuous variables based on their normality or non-normality distribution. Parametric statistics (t-tests and analysis of variance) were used on normally distributed data to test for differences in baseline pulmonary function, the change in FEV1/FVC, and IgE between the two groups. And non-parametric statistics (the Mann-Whitney, and Kruskal-Wallis tests) were used if the distribution deviated from normal to identify the influence of genotypes on EOS and Δ FEV1. Bonferroni correction was used for pairwise comparisons. The count data were expressed using case number and constituent ratio (%) and the groups’ comparison using the Chi-Square test. Fisher’s exact test and Pearson’s chi-square test were used to test the significant correlation between SNVs and ICS response. Genotypic distribution, allelic frequencies, and haplotypes were compared by unconditional logistic regression with a priori adjustments for biological sex, age, BMI, ETS exposure, and residence. Afterward, odds ratios (ORs), and 95% confidence intervals (CIs) were calculated, and association *p*-values estimated. The interaction between the *GAB1* gene and the environment was analysed using the generalized multifactor dimensionality reduction (GMDR) method. A statistically significant difference was defined as *p*<0.05.

## Results

### Baseline characteristics

Table 2 summarizes the baseline characteristics of participants. The asthma group included 109 children, 77 boys and 32 girls, aged 2 to 15 years. The control group included 158 children, 99 boys and 59 girls, 2 to 15 years. The distribution of biological sex, age, feeding behaviors, and pet exposure did not differ significantly between the two groups (*p* > 0.05); the BMI was higher in the asthma group than in the control group (16.07 vs 15.74, *p* = 0.015). Significant differences existed between groups in allergen exposure, ETS exposure, and residence (*p* < 0.05). Specifically, asthma patients presented a greater percentage of exposure to allergens and ETS, as well as living more frequently in urban areas. All asthmatics received ICS treatment. Among 74 asthmatics aged at least 5, 71 completed the 12-week follow-up, whereas the others did not or received substandard treatment.

**Table 2:** Baseline characteristics of the subjects

Description	Asthmatics	Controls	*p*
**Anthropometry**			
Biological sex, n (%)			
Males	77(70.64)	99(62.66)	0.176
Females	32(29.36)	59(37.34)	
Age**,** median (IQR),year	6(4,8)	6(4,8)	0.383
BMI, median (IQR), kg/m^2^	16.07(15.17,18.53)	15.74(12.13,16.66)	**0.015**
Exposure factors			
Feeding patterns, n (%)			
Artificial/mixed feeding	86(78.90)	129(81.65)	0.578
Breastfeeding	23(21.10)	29(18.35)	
Allergen exposure, n (%)			
Yes	75(68.81)	15(9.49)	**< 0.001**
No	34(31.19)	143(90.51)	
ETS exposure, n (%)			
Yes	29(26.61)	9(5.70)	**< 0.001**
No	80(73.39)	149(94.30)	
Pet exposure, n (%)			
Yes	27(24.77)	34(21.52)	0.534
No	82(75.23)	124(78.48)	
Residence, n (%)			
Urban	75(68.81)	83(52.53)	**0.008**
Rural	34(31.19)	75(47.47)	
Laboratory indices			
EOS(%)	5.00 ± 5.52		NA
IgE(IU/ml)	256.86 ± 390.27		NA
Spirometry			
FVC% pred^a^	92.12 ± 22.30		NA
FEV1 (%)^a^	83.90 ± 17.59		NA
FEV1/FVC^a^	78.87 ± 12.28		NA
PEF% pred^a^	68.11 ± 18.41		NA
FEF25% pred^a^	66.36 ± 20.94		NA
FEF50% pred^a^	61.44 ± 24.49		NA
FEF75% pred^a^	52.46 ± 26.90		NA
MMEF% pred^a^	58.50 ± 24.55		NA

Comparison of biological sex and exposure factors between two groups used χ^2^ test, while age and BMI used Mann-Whitney test. The values *p* < 0.05 were in bold

Abbreviations: *ETS* environmental tobacco smoke, *BMI* body mass index

^a^Baseline pulmonary function of asthmatic children aged ≥5 years

### Analysis of genotype and allele distribution

The genotype distribution of all *GAB1* SNVs in the control group (rs1397527, rs17017425, rs1866756, rs3805236, rs3805246, and rs3805254) was in HWE (*p* > 0.05). There was no significant difference in the distribution of *GAB1* genotypes and alleles between asthma and control groups (*p* > 0.05). Details of these results are shown in Table 3.

**Table 3:** Association between *GAB1* gene variants and asthma risk

SNVs	Genotypes/ Alleles	Asthma (*n* = 109)	Control (*n* = 158)	*p*^a^	OR (95%CI)	*p*^b^
rs1396527						
	G/G	47(43.1)	85(53.8)	0.102	–	–
	G/T	55(50.5)	59(37.3)		1.57(0.88–2.79)	0.125
	T/T	7(6.4)	14(8.9)		0.78(0.27–2.31)	0.658
	G	149(68.4)	229(72.5)	0.304	–	–
	T	69(31.7)	87(27.5)		1.41(0.81–2.45)	0.222
rs17017425						
	T/T	55(50.5)	89(56.3)	0.573	–	–
	T/C	47(43.1)	58(36.7)		1.08(0.61–1.92)	0.782
	C/C	7(6.4)	11(7.0)		1.18(0.41–3.45)	0.760
	T	157(72.0)	236(74.7)	0.492	–	–
	C	61(28.0)	80(25.3)		1.10(0.64–1.89)	0.736
rs1866756						
	G/G	57(52.3)	92(58.2)	0.287	–	–
	G/A	48(44.0)	56(35.4)		1.32(0.74–2.33)	0.346
	A/A	4(3.7)	10(6.3)		0.64(0.17–2.35)	0.501
	G	162(74.3)	240(76.0)	0.666	–	–
	A	56(25.7)	76(24.1)		1.21(0.70–2.10)	0.503
rs3805236						
	A/A	54(49.5)	90(57.0)	0.248	–	–
	A/G	48(44.0)	54(34.2)		1.42(0.79–2.54)	0.241
	G/G	7(6.4)	14(8.9)		0.85(0.30–2.43)	0.761
	A	156(71.6)	234(74.1)	0.524	–	–
	G	62(28.4)	82(26.0)		1.30(0.75–2.26)	0.359
rs3805246						
	G/G	58(53.2)	92(58.2)	0.342	–	–
	G/A	47(43.1)	56(35.4)		1.28(0.72–2.26)	0.401
	A/A	4(3.7)	10(6.3)		0.63(0.17–2.32)	0.488
	G	163(74.8)	240(76.0)	0.756	–	–
	A	55(25.2)	76(24.1)		1.17(0.68–2.04)	0.568
rs3805254						
	A/A	58(53.2)	92(58.2)	0.342	–	–
	A/G	47(43.1)	56(35.4)		1.28(0.72–2.26)	0.401
	G/G	4(3.7)	10(6.3)		0.63(0.17–2.32)	0.488
	A	163(74.8)	240(76.0)	0.756	–	–
	G	55(25.2)	76(24.1)		1.17(0.68–2.04)	0.568

Abbreviations: *OR* odds ratio, *CI* confidence interval, ^*a*^ *χ*^*2*^ test for genotype and allele distributions between the asthma group and the control group; ^b^ binary logistic regression for genotype distributions between the asthma group and the control group, adjusted for biological sex, age, BMI, ETS exposure and residence

### Analysis of haplotypes and linkage disequilibrium (LD)

**Fig. 1:**
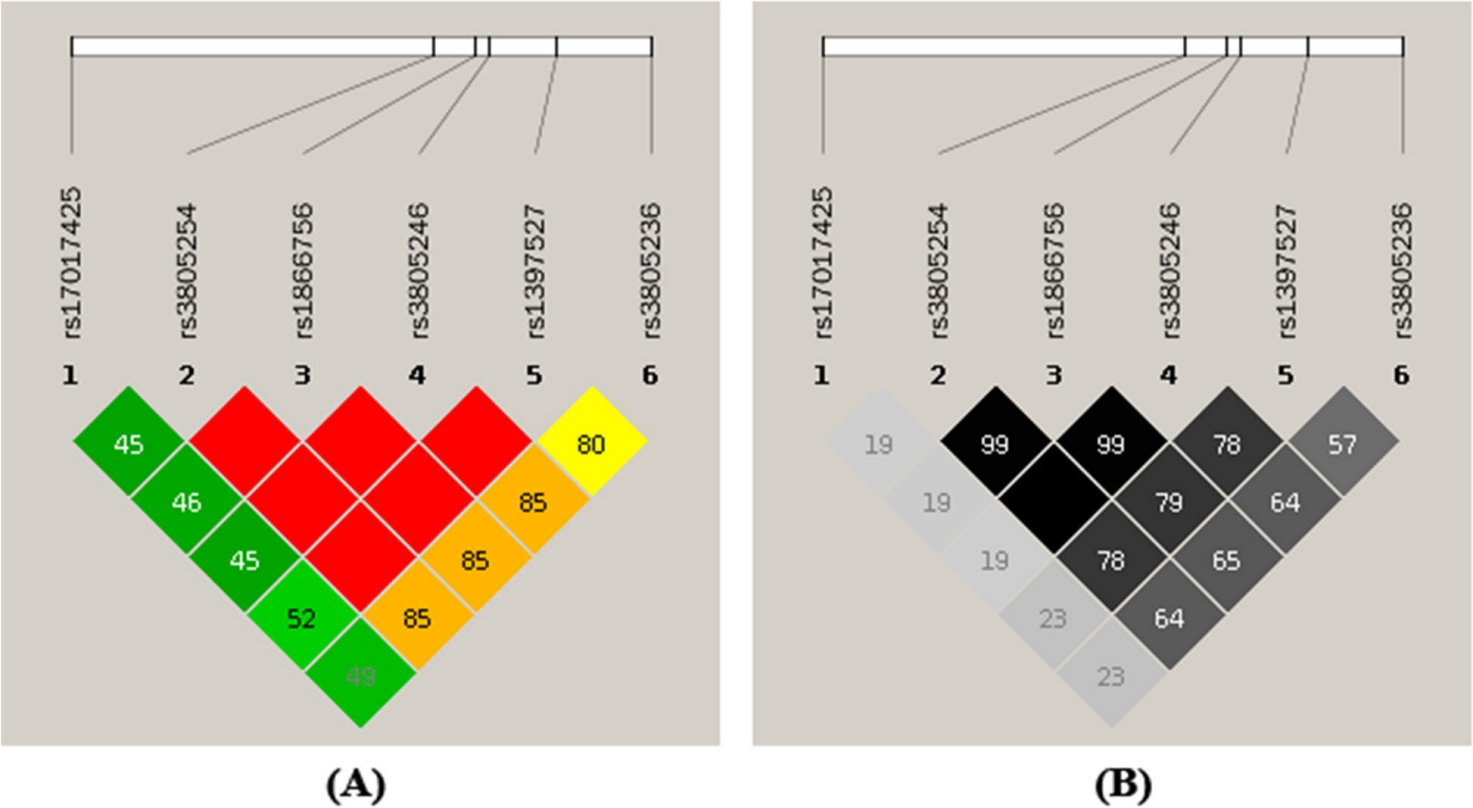
Linkage disequilibrium diagram. (**A**) coefficient of LD (D′). The D′ value is displayed in gradients from red (strong linkage) to green (weak linkage). (**B**) correlation coefficient (r^2^). The r^2^ value is displayed in gradients from black (strong linkage) to gray (weak linkage)

Haploview software was used to examine haplotype and LD analyses. Based on Fig. 1, all of the SNVs were in high LD except rs17017425 which was physically distant from the rest of the SNVs. Meanwhile, haplotype analysis revealed that the *GAB1* six loci (rs3805236-rs3805246-rs1397527-rs3805254-rs1866756-rs17017425) composed five haplotypes. AGGAGT was the most frequent haplotype in the asthma control group (65.19%) (Table 4). Following statistical analysis, the asthma group had a greater frequency of haplotype AGGAGC in comparison to the control group using haplotype AGGAGT as the reference (*p* = 0.030). Using a logistic regression model adjusted by several confounders, those results were mimicked, being the haplotype AGGAGC associated with a statistically increased risk for asthma susceptibility (OR = 2.69, *p* = 0.018).

**Table 4:** Haplotype analysis for blocks in *GAB1* gene

Haplotype	Asthma (*n* = 197)	Control (*n* = 296)	*P*^*a*^	OR (95%CI)	*P*^*b*^
AGGAGC	18(8.26)	12(3.80)	**0.030**	2.69(1.19–6.07)	**0.018**
AGGAGT	123(56.42)	206(65.19)	0.458	–	–
GATGAC	25(11.47)	44(13.92)	0.553	0.88(0.48–1.59)	0.669
GATGAT	25(11.47)	24(7.59)	0.133	1.74(0.90–3.36)	0.101
GGGAGC	6(2.75)	10(3.16)	0.843	1.05(0.35–3.19)	0.930

n refers to the number of haplotypes (frequency > 3%)

The values *p* < 0.05 were in bold

^a^calculated with χ^2^ test; ^b^ calculated with logistic regression test adjusted for biological sex, age, BMI, ETS exposure, and residence

### Association between the *GAB1* gene variants and EOS and IgE

The Kruskal-Wallis H test revealed that percentage of EOS and genotype distribution of rs1397527 were associated (*p* = 0.007). Pairwise comparisons showed that GG and GT genotypes were statistically different in terms of EOS percentage, with the EOS percentage lower in GG genotype in comparison with GT genotype (3.00 vs 5.50, Bonferroni-adjusted *p* = 0.005). No statistically significant differences were observed for EOS percentage or IgE levels between the other genotypes or alleles (Table 5).

**Table 5:** Association results between genetic variants in *GAB1* gene, and percentage of EOS and IgE levels

SNVs	Genotypes	EOS (%)	*p*^a^	IgE(IU/ml)	*p*^b^
rs1396527					
	G/G	3.00(1.30–5.60)	**0.007**	2.08 ± 0.60	0.848
	G/T	5.50(2.50–7.70)		2.14 ± 0.51	
	T/T	3.70(2.20–6.70)		2.18 ± 0.46	
rs17017425					
	T/T	3.10(1.60–6.40)	0.102	2.04 ± 0.50	0.390
	C/T	4.80(2.20–7.60)		2.20 ± 0.58	
	C/C	6.80(3.90–8.30)		2.12 ± 0.59	
rs1866756					
	G/G	3.30(1.60–6.20)	0.136	2.09 ± 0.58	0. 846
	G/A	4.80(2.60–7.18)		2.14 ± 0.50	
	A/A	2.40(0.63–11.53)		2.22 ± 0.58	
rs3805236					
	A/A	3.50(1.60–6.15)	0.309	2.09 ± 0.58	0.877
	A/G	4.45(2.40–7.18)		2.15 ± 0.52	
	G/G	4.80(2.50–14.50)		2.10 ± 0.41	
rs3805246					
	G/G	3.50(1.60–6.48)	0.240	2.10 ± 0.58	0. 064
	G/A	4.80(2.50–6.80)		2.13 ± 0.50	
	A/A	2.40(0.63–11.53)		2.22 ± 0.58	
rs3805254					
	A/A	3.50(1.60–6.48)	0.240	2.10 ± 0.58	0. 064
	A/G	4.80(2.50–6.80)		2.13 ± 0.50	
	G/G	2.40(0.63–11.53)		2.22 ± 0.58	

The values *p* < 0.05 were in bold

Abbreviations: ^a^ Kruskal-Wallis H test for EOS distribution between genotypes in the asthma group; ^b^ ANOVA for lg (lgE) distribution between genotypes in the asthma group

### Relationship between SNVs and baseline pulmonary function

We analysed the relationship between variants in *GAB1* gene and pulmonary function indices before ICS treatment including FVC% pred, FEV1% pred, FEV1/FVC, PEF% pred, FEF25% pred, FEF50% pred, FEF75% pred, MMEF% pred, and we found no significant association with the *GAB1* SNVs before the 12-week ICS treatment. Baseline pulmonary function indices of the genotypes are revealed in the supplementary file (Table S1).

### Analysis of the changes in FEV1/FVC and Δ FEV1 after ICS treatment

**Fig. 2:**
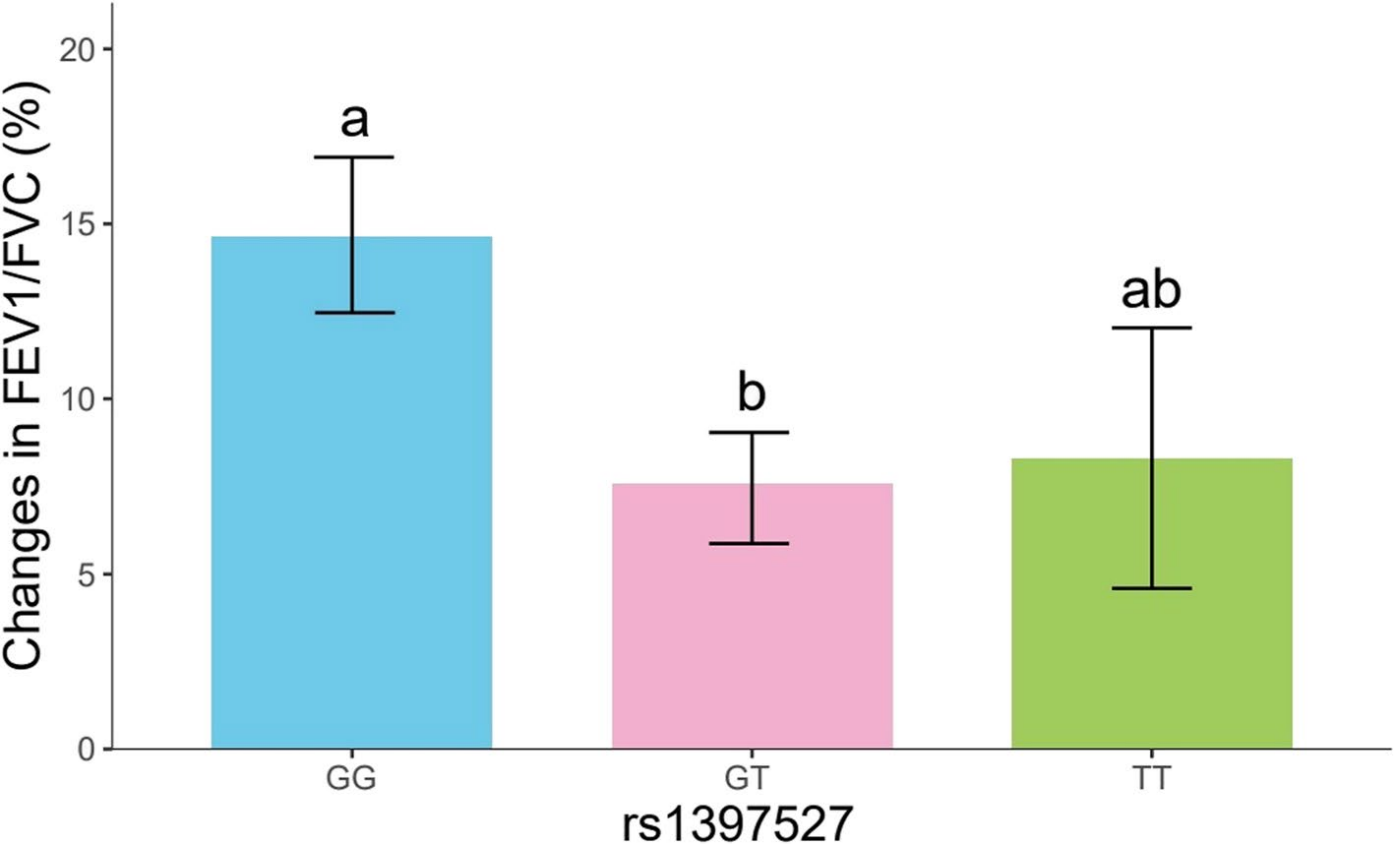
Changes in FEV1/FVC for the rs1397527 genotypes after treatment. The letters a and b appear separately to indicate differences between groups and there is no difference between groups with same letter

We analysed the association between *GAB1* SNVs and changes in FEV1/FVC after 12-weeks of ICS treatment (Table 6). We found significant differences in the distribution of the FEV1/FVC change at rs1397527 (*p* = 0.032). Figure 2 demonstrates the change in FEV1/FVC of each genotype at rs1397527. Further comparisons revealed that the ratio was higher in the GG genotype group than GT genotype group, precisely 14.84% vs 7.93% (adjusted *p* < 0.05). Furthermore, we explored each genotype’s dominant and recessive models (Table 6). The dominant model showed that the change in FEV1/FVC of rs1397527 minor homozygotes (TT) and heterozygotes (GT) was lower than that in reference homozygotes (GG) after ICS treatment (*p* = 0.009). Moreover, children with rs3805236 reference homozygous (AA) showed higher percentage change in FEV1/FVC compared with the minor genotype (GG/AG) (*p* = 0.025). However, this phenomenon was not repeated in the other gene loci.

**Table 6:** Association between *GAB1*gene SNVs and change in FEV1/FVC after ICS treatment

SNVs	Genotypes	Change in FEVI/FVC (%)	*p*	*p*^a^	*p*^b^
rs1396527					
	G/G	14.84 ± 12.13	**0.032**	**0.009**	0.593
	G/T	7.93 ± 9.49			
	T/T	8.31 ± 8.31			
rs17017425					
	T/T	12.26 ± 8.94	0.366	0.220	0.270
	C/T	9.71 ± 14.08			
	C/C	6.10 ± 10.13			
rs1866756					
	G/G	12.89 ± 12.14	0.273	0.109	0.585
	G/A	8.80 ± 9.50			
	A/A	7.44 ± 9.50			
rs3805236					
	A/A	13.75 ± 11.51	0.061	**0.025**	0.262
	A/G	8.26 ± 9.99			
	G/G	2.21 ± 4.05			
rs3805246					
	G/G	12.89 ± 12.14	0.273	0.109	0.585
	G/A	8.80 ± 9.50			
	A/A	7.44 ± 9.50			
rs3805254					
	A/A	12.89 ± 12.14	0.273	0.109	0.585
	A/G	8.80 ± 9.50			
	G/G	7.44 ± 9.50			

The values *p* < 0.05 were in bold

^a^ dominant model; ^b^ recessive model

We also analysed the association between *GAB1* SNVs and Δ FEV1. Whereas, this analysis failed to show an association, which is provided in the supplementary file (Table S2).

### Analysis of ICS response

The general characteristics of ICS responders and nonresponders are shown in the supplementary file (Table S3). There was no significant difference between the two groups in age, gender, BMI, or baseline pulmonary function. The dominant model of rs1397527 was associated with ICS response after 12-weeks treatment (*p* = 0.038). Specifically, asthmatics carrying the GG genotype had better treatment responses than patients carrying the GT or TT genotype (Table 7).

**Table 7:** Association between *GAB1* gene SNVs and ICS response

SNVs	Genotypes	ICS responders	ICS nonresponders	*p*	*p*^a^	*p*^b^
rs1396527						
	G/G	22(52.4)	8(27.6)	**0.036**	**0.038**	0.642
	G/T	16(38.1)	20(69.0)			
	T/T	4(9.5)	1(3.4)			
rs17017425						
	T/T	25(59.5)	16(55.2)	0.760	0.715	> 0.999
	T/C	13(31.0)	11(37.9)			
	C/C	4(9.5)	2(6.9)			
rs1866756						
	G/G	23(54.8)	14(48.3)	0.283	0.591	0.265
	G/A	16(38.1)	15(51.7)			
	A/A	3(7.1)	0(0.0)			
rs3805236						
	A/A	22(52.4)	14(48.3)	0.542	0.734	0.510
	A/G	18(42.9)	15(51.7)			
	G/G	2(4.8)	0(0.0)			
rs3805246						
	G/G	23(54.8)	14(48.3)	0.230	0.591	0.265
	G/A	16(38.1)	15(51.7)			
	A/A	3(7.1)	0(0.0)			
rs3805254						
	A/A	23(54.8)	14(48.3)	0.230	0.591	0.265
	A/G	16(38.1)	15(51.7)			
	G/G	3(7.1)	0(0.0)			

The values *p* < 0.05 were in bold

^a^ dominant model; ^b^ recessive model

### SNV-SNV interaction

**Fig. 3:**
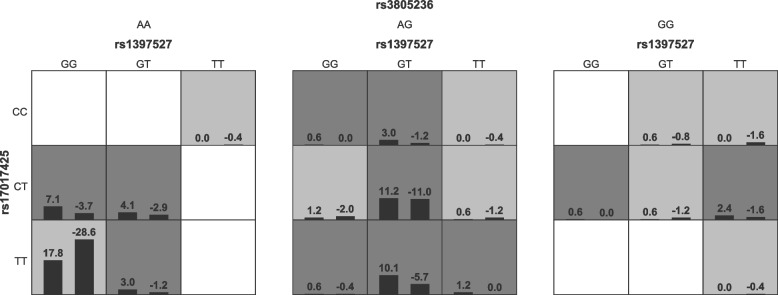
Analysis of the three-order model. Each cell represents an interaction combination; highrisk cells are denoted by a dark color, lowrisk cells are denoted by a light tint. The more positive the score in the cell, the higher the risk of asthma in the combo

GMDR analysis was performed to assess the effect of SNV-SNV interactions on asthma risk. The results exhibited that rs1397527 was an important SNV in all models. The three-order model (rs1397527, rs17017425, and rs3805236) was the best model after permutation testing. The three-order model had the best cross-validation consistency of 10/10 and the highest testing accuracy of 0.564, after adjusting for biological sex, age, and BMI, there was a trend that the model was associated with asthma (*p* = 0.055, Table 8). As shown in Fig. 3, when the genotype of rs1397527 was GT, rs3805236 was AG, and rs17017425 was TT, the risk of asthma was highest in the three-order model.

**Table 8:** GMDR analysis of *GAB1* SNVs

Model	Testing accuracy	Cross-validation consistency	*p*
rs1397527/rs17017425	0.552	7/10	0.055
rs1397527/rs17017425/rs3805236	0.564	10/10	0.055
rs1397527/rs17017425/ rs3805236/rs3805246	0.536	8/10	0.377
rs1397527/rs17017425/rs1866756/rs3805236/rs3805246	0.537	8/10	0.377
rs1397527/rs17017425/rs1866756/rs3805236/rs3805246/rs3805254	0.537	10/10	0.377

Adjusted for biological sex, age, and BMI

### Gene-environment interaction

**Fig. 4:**
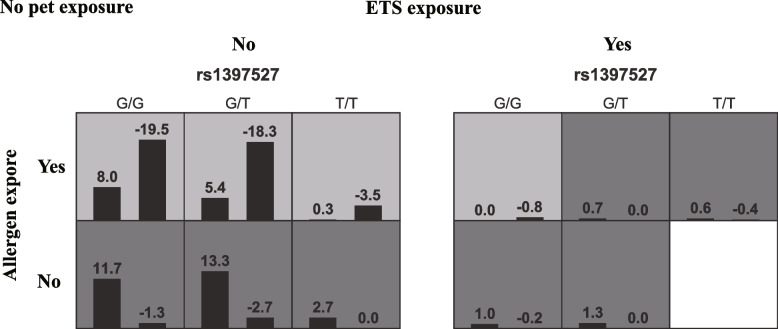
Analysis of the four-order model. Each cell represents an interaction combination; high-risk cells are denoted by a dark color, low-risk cells are denoted by a light tint. The more positive the score in the cell, the higher the risk of asthma in the combo

In this study, the rs1397527 locus of the *GAB1* gene was associated with EOS and ICS efficacy and was more strongly associated with asthma than the other loci. Therefore, the GMDR method was used to analyze the interaction between rs1397527 locus and feeding patterns, allergen exposure, ETS exposure, pet exposure, and residence. As shown in Fig. 4, a significant four-order model revealed a potential gene-environment interaction among rs1397527, allergen exposure, ETS exposure, and pet exposure with the best cross-validation consistency (10/10) and testing accuracy of 0.821 (*p* = 0.001, Table 9).

**Table 9:** GMDR analysis of *GAB1* rs1397527 and environmental variables

Model	Testing accuracy	Cross-validation consistency	*p*
rs1397527/Allergen exposure/ETS exposure	0.818	10/10	0.001
rs1397527/Allergen exposure/ETS exposure/Pet exposure	0.821	10/10	0.001
rs1397527/Allergen exposure/ETS exposure/Pet exposure/Residence	0.819	10/10	0.001
rs1397527/Feeding patterns/Allergen exposure/ETS exposure/Pet exposure /Residence	0.810	10/10	0.001

Adjusted for biological sex, age and BMI

## Discussion

Asthma is a chronic heterogeneous airway disorder with a major genetic component. With the advancement of genetic and molecular biology research, genes have been increasingly identified as directly associated with asthma [4]. Therefore, genetic susceptibility is critical for diagnosing and screening asthma. *GAB1* is the most highly expressed and broadly expressed member of the growth factor receptor-related binding protein (Gab) family and is found on chromosome 4q31.21. It can mediate cell signaling through tyrosine phosphatase SHP2 or phosphatidylinositol-3 kinase (PI3K) in response to various extracellular stimuli [21]. Wang et al. [22] discovered that *GAB1* was expressed in AT-IIs and that the depletion of *GAB1* in AT-IIs caused an imbalance in surfactant protein synthesis, the appearance of disorganized lamellar bodies and predisposed susceptibility to lung injury. This suggests that *GAB1* is critical in preserving the homeostasis and functional integrity of AT-IIs in the alveoli. Asthma is an inflammatory illness of the airways primarily mediated by T helper type-2 (Th2) inflammation cells. Dendritic cells (DCs) play an essential role in the development of primary immune and allergic reactions by presenting antigens to Th2 cells. Zhang et al. [23] reported the significance of *GAB1* in regulating DC migration in allergic asthma. They observed an elevation of *GAB1* level in peripheral blood mononuclear cells from asthmatic patients during acute exacerbation, and further experiments found that *GAB1* can regulate DC migration in allergic asthma by affecting CCL19/CCR7 signaling. Therefore, *GAB1* may be involved in the pathogenesis of asthma.

According to the currently reported genome-wide association studies (GWAS), the LD block region including rs1397527 and containing the USP38/*GAB1* genes has been identified as a susceptibility locus for adult asthma in the Japanese population [16]. However, there was no association between *GAB1* rs1397527 and susceptibility to childhood asthma in our study. We speculate that the unexpected results are attributable to not considering all the variants that correlated by LD within the region. Besides, the relatively small sample size of this study results in lower statistical power. A study targeting childhood asthma in Europeans found that the variant of the *GAB1* rs3805236 was associated with susceptibility to childhood asthma [24]. By contrast, in the current study no difference in the distribution of genotypes and alleles of rs3805236 between asthma and control groups were discovered, implying that rs3805236 may not represent a susceptibility locus for asthma in Chinese children, and we consider that it might be affected by regional ethnicity. Previous studies have validated the association of rs3805246 with meningioma, cholangiocarcinoma, and gastritis [25–27]. However, there is currently no correlation study between this locus and childhood asthma, and our study revealed no link between this locus and childhood asthma susceptibility. In addition, for the rs17017425, rs1866756, and rs3805254, we did not find any association with increased susceptibility and risk of childhood asthma.

Following the rationale that combining alleles into haplotypes according to LD could depict the genuine link between genes and diseases compared to individual SNV analysis [28], we carried out haplotype association analyses. Although no link was observed between asthma and any of the *GAB1* SNVs studied, haplotype analysis revealed that haplotype AGGAGC was linked to childhood asthma susceptibility and was a risk factor for asthma.

Our study found an association between *GAB1* rs1397527 and EOS. The results showed that the EOS levels were higher in asthmatic children carrying GT genotype than GG genotype, indicating that the increase in EOS may be connected to the presence of the minor allele T in rs1397527. *GAB1* can mediate cell signaling through tyrosine phosphatase SHP2. Accumulating evidence indicates that SHP2 plays a crucial regulatory role in the differentiation of eosinophils. Mice with bone marrow SHP2 deficiency have been demonstrated to be unable to induce eosinophilia and airway hyperresponsiveness [29]. It could be hypothesized that indirectly activated SHP2 because of the presence of the minor allele T results in eosinophilia.

ICS is the most effective treatment for childhood asthma, which can improve asthma symptoms, pulmonary function, airway hyperresponsiveness, and the frequency of acute attacks. Nevertheless, there is significant heterogeneity in ICS therapeutic effects and adverse reactions [30]. Under standardized use, some children’s symptoms may be unable to be alleviated or even worsen despite an increasing dosage [31]. Glucocorticoid (GC) pharmacogenomics research and GWAS have confirmed that genetic variation is associated with steroid treatment response [32]. Sharma et al. [15] observed that *GAB1* levels in BEAS-2B bronchial epithelial cells increased after GC treatment, and siRNA knockdown of *GAB1* in BEAS-2B cells decreased the expression of NF-κB. This finding demonstrated that *GAB1* could positively regulate the presentation of the pro-inflammatory factor NF-κB in asthma through certain pathways. The NF-κB signaling pathway, as is well known, can be transcribed through TNF- α, IL-6, IL-12, and other cytokines [33], which mediate the immunological response and participate in the signaling pathways that GC affects [34]. Therefore, we speculate that *GAB1* is a response gene for GC therapy of asthma. To verify this hypothesis, we evaluated the change in pulmonary function after ICS treatment among different genotypes of *GAB1* SNVs, and variants of rs1397527 and rs3805236 were found to be associated with the change in FEV1/FVC after therapy. In particular, we discovered that minor alleles for *GAB1* rs1397527 and rs3805236 had less improvement in FEV1/FVC compared with reference homozygotes. Likewise, for ICS response, asthmatics with rs1397527 minor alleles (GT or TT genotypes) had a worse therapeutic response than subjects carrying the GG genotype. These pieces of evidence could be inferred that minor alleles of rs1397527 and rs3805236 may boost *GAB1* expression, hence increasing the activity of NF-κB and antagonizing the anti-inflammatory effect of ICS, although there was no relevant functional data. It was also confirmed by the GTEx portal and PhenoScanner databases that the minor alleles at rs1397527 and rs3805236 were effect alleles and were associated with the expression of GAB1 gene, USP38 gene, and GUSBP5 gene. Interestingly, NF-κB is the central inflammatory factor in severe eosinophilic asthma [34, 35]. Blood eosinophilia can be used as one of the indicators to evaluate the poor efficacy of ICS anti-inflammatory treatment [36], which is consistent with the discovery in this study that carrying the minor allele T at rs1397527 can cause eosinophilia.

Asthma is a multifactorial disease impacted by various environmental factors, polygenes, and their interactions. Interaction is the combination of multiple factors, leading to a certain outcome. Particular gene variants determine disease risk following environmental exposure, known as gene-environment interactions [37]. Particular combinations of SNVs determine disease risk, known as SNV-SNV interactions. The complexities of asthma necessitate investigating how genes are expressed or suppressed in specific settings. Previously, feeding patterns, allergen exposure, ETS exposure, pet exposure, and residence have been confirmed to be important risk factors for asthma [38–42]. In this study, the GMDR was used for interaction detection, and the results showed the interaction of rs1397527, allergen, ETS, and Pet exposure, suggesting that SNV and specific environments could interact and improve the prediction of asthma. Furthermore, we also found a trend that interaction of rs1397527, rs17017425, and rs3805236.

To our knowledge, this is the first study to report the relationship between *GAB1* gene SNVs and asthma and ICS efficacy. However, our study has several limitations. First of all, limited number of SNVs in the *GAB1* gene were included in the current study, and more SNVs should be included in the analysis in the future. Second, the diagnosis of asthma in children under 5 years mainly depended on symptoms and signs, and the lack of spirometry could cause a bias in the diagnosis of asthma in the very early years of life. Furthermore, the basic characteristics of the control group and the asthma group populations were different, and the potential impact of these factors on the results could not be completely eliminated after adjusting for statistical analysis. Third, for the study of ICS efficacy, we only used Δ FEV1 and the change in FEV1/FVC as the primary outcomes, but other indexes, such as PEF, should be included in further studies. Moreover, we notice that baseline pulmonary function indexes may be imbalanced between ICS responders and nonresponders, and the improvement in spirometry in ICS nonresponders after treatment may be restricted by their higher baseline level. For the true relationship between the expression of GABI gene and the activity of NF-κB, further functional validation should be conducted to better understand its role in ICS treatment of asthma. Finally, the number of children in the study is small, so our results should be interpreted with caution, and larger sample sizes are needed to confirm our findings.

## Conclusions

**Fig. 5:**
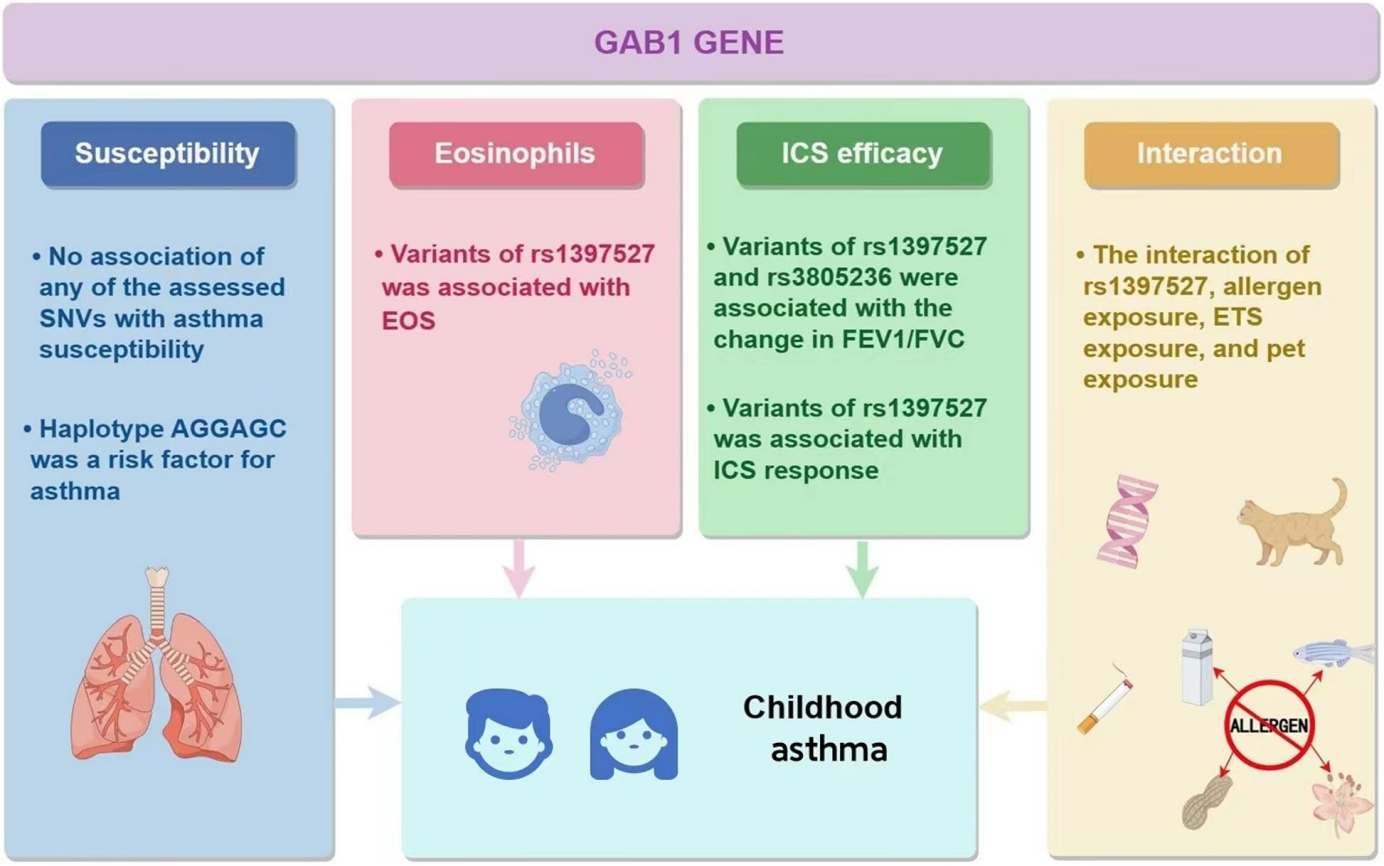
Main findings of the current study

Collectively, this study analysed the genetic variant of the *GAB1* gene and found that no SNVs were individually associated with asthma susceptibility, but when analyzing haplotypes of variants located in GAB1 gene there were positive results that haplotype AGGAGC was a risk factor for asthma. And *GAB1* SNVs were associated with EOS and ICS efficacy. In addition, we also observed gene-environment interaction, providing a theoretical basis for studying genetic-related pathogenesis and predicting ICS efficacy and individualized treatment in Chinese children with asthma. Figure 5 highlights the study’s main findings. In addition, other than asthma, our research also provides a new direction of gene-target therapy for the diagnosis and treatment of other GC-treated diseases. In the future, relevant functional verification and research on larger sample sizes, different regions, and ethnic groups should be carried out to validate our results.

### Supplementary Information

**Additional file 1.**

## Data Availability

The data presented in the study are deposited in the EVA repository (https://www.ebi.ac.uk/eva/?eva-study=PRJEB63147). Further inquiries can be directed to the corresponding author.

## References

[1] Holgate ST, Wenzel S, Postma DS, Weiss ST, Renz H, Sly PD (2015). Asthma Nat Rev Dis Primers..

[2] Tai A, Tran H, Roberts M, Clarke N, Gibson AM, Vidmar S (2014). Outcomes of childhood asthma to the age of 50 years. J Allergy Clin Immunol..

[3] The global asthma report 2022. Int J Tuberc Lung Dis. 2022;26(1):1–104. 10.5588/ijtld.22.1010 36303302

[4] El-Husseini ZW, Gosens R, Dekker F, Koppelman GH (2020). The genetics of asthma and the promise of genomics-guided drug target discovery. Lancet Respir Med..

[5] Polderman TJ, Benyamin B, de Leeuw CA, Sullivan PF, van Bochoven A, Visscher PM (2015). Meta-analysis of the heritability of human traits based on fifty years of twin studies. Nat Genet..

[6] Paciência I, Cavaleiro Rufo J, Moreira A. Environmental inequality: air pollution and asthma in children. Pediatr Allergy Immunol. 2022;33(6) 10.1111/pai.13818. 10.1111/pai.13818 35754123

[7] Miller RL, Lawrence J (2018). Understanding root causes of asthma. Perinatal environmental exposures and epigenetic regulation. Ann Am Thorac Soc..

[8] Kew KM, Flemyng E, Quon BS, Leung C (2022). Increased versus stable doses of inhaled corticosteroids for exacerbations of chronic asthma in adults and children. Cochrane Database Syst Rev..

[9] Dijkstra A, Postma DS, Bruinenberg M, van Diemen CC, Boezen HM, Koppelman GH (2011). SERPINE1 -675 4G/5G polymorphism is associated with asthma severity and inhaled corticosteroid response. Eur Respir J..

[10] Cazzola M, Rogliani P, Calzetta L, Matera MG (2020). Pharmacogenomic response of inhaled corticosteroids for the treatment of asthma: considerations for therapy. Pharmgenomics Pers Med..

[11] Szalai C, Ungvári I, Pelyhe L, Tölgyesi G, Falus A (2008). Asthma from a pharmacogenomic point of view. Br J Pharmacol..

[12] Athari SS (2019). Targeting cell signaling in allergic asthma. Signal Transduct Target Ther..

[13] Wilson SJ, Wallin A, Della-Cioppa G, Sandström T, Holgate ST (2001). Effects of budesonide and formoterol on NF-kappaB, adhesion molecules, and cytokines in asthma. Am J Respir Crit Care Med..

[14] Gagliardo R, Chanez P, Mathieu M, Bruno A, Costanzo G, Gougat C (2003). Persistent activation of nuclear factor-kappaB signaling pathway in severe uncontrolled asthma. Am J Respir Crit Care Med..

[15] Amitabh S, Jörg M, Chris HC, Tatiana O, Xiaobo Z, Maksim K, et al. A disease module in the interactome explains disease heterogeneity, drug response and captures novel pathways and genes in asthma. Hum Mol Genet. 2015;24(11) 10.1093/hmg/ddv001 PMC4447811 25586491

[16] Hirota T, Takahashi A, Kubo M, Tsunoda T, Tomita K, Doi S (2011). Genome-wide association study identifies three new susceptibility loci for adult asthma in the Japanese population. Nat Genet..

[17] Guideline for the diagnosis and optimal management of asthma in children(2016). Zhonghua Er Ke Za Zhi. 2016;54(3):167–81. 10.3760/cma.j.issn.0578-1310.2016.03.003. 10.3760/cma.j.issn.0578-1310.2016.03.003 26957061

[18] Hernandez-Pacheco N, Farzan N, Francis B, Karimi L, Repnik K, Vijverberg SJ (2019). Genome-wide association study of inhaled corticosteroid response in admixed children with asthma. Clin Exp Allergy..

[19] Tse SM, Gold DR, Sordillo JE, Hoffman EB, Gillman MW, Rifas-Shiman SL (2013). Diagnostic accuracy of the bronchodilator response in children. J Allergy Clin Immunol..

[20] Neelamegan R, Saka V, Tamilarasu K, Rajaram M, Selvarajan S, Chandrasekaran A (2016). Clinical utility of fractional exhaled nitric oxide (FeNO) as a biomarker to predict severity of disease and response to inhaled corticosteroid (ICS) in asthma patients. J Clin Diagn Res..

[21] Do DC, Agrawal A, Luo X, Gao P. Gab1, a therapeutic target for allergic asthma? J Xiangya Med. 2017;2 10.21037/jxym.2017.03.02. 10.21037/jxym.2017.03.02 PMC6105283 30148256

[22] Wang K, Qin S, Liang Z, Zhang Y, Xu Y, Chen A (2016). Epithelial disruption of Gab1 perturbs surfactant homeostasis and predisposes mice to lung injuries. Am J Physiol Lung Cell Mol Physiol..

[23] Yun Z, Yun X, Shuwan L, Xiaohong G, Dong C, Jiaqi X, et al. Scaffolding protein Gab1 regulates myeloid dendritic cell migration in allergic asthma. Cell Res. 2016;26(11) 10.1038/cr.2016.124 PMC5099873 27811945

[24] Li L, Kabesch M, Bouzigon E, Demenais F, Farrall M, Moffatt MF (2013). Using eQTL weights to improve power for genome-wide association studies: a genetic study of childhood asthma. Front Genet..

[25] Chen W, Zhao J, Wu Q, Yan H, Wang X, Nan C (2021). Association of Single-Nucleotide Polymorphisms of Gab1 Gene with susceptibility to meningioma in a northern Chinese Han population. Med Sci Monit..

[26] Gao L, Weck MN, Nieters A, Brenner H (2010). Grb2-associated binder 1 (Gab1) genetic polymorphism, helicobacter pylori infection, and chronic atrophic gastritis among older adults from Germany. Mol Carcinog..

[27] Meng LQ (2014). Essential role of polymorphism of Gab1, EGFR, and EGF for the susceptibility of biliary tract cancer. Tumour Biol..

[28] Crawford DC, Nickerson DA (2005). Definition and clinical importance of haplotypes. Annu Rev Med..

[29] Qin XJ, Zhang GS, Zhang X, Qiu ZW, Wang PL, Li YW (2012). Protein tyrosine phosphatase SHP2 regulates TGF-β1 production in airway epithelia and asthmatic airway remodeling in mice. Allergy..

[30] Ramamoorthy S, Cidlowski JA (2016). Corticosteroids: mechanisms of action in health and disease. Rheum Dis Clin N Am..

[31] Guilbert TW, Bacharier LB, Fitzpatrick AM (2014). Severe asthma in children. J Allergy Clin Immunol Pract..

[32] Daya M, Ortega VE (2020). Asthma genomics and pharmacogenomics. Curr Opin Immunol..

[33] Mohan RR, Mohan RR, Kim WJ, Wilson SE (2000). Modulation of TNF-alpha-induced apoptosis in corneal fibroblasts by transcription factor NF-kappaB. Invest Ophthalmol Vis Sci..

[34] Edwards MR, Bartlett NW, Clarke D, Birrell M, Belvisi M, Johnston SL (2009). Targeting the NF-kappaB pathway in asthma and chronic obstructive pulmonary disease. Pharmacol Ther..

[35] Yang L, Cohn L, Zhang DH, Homer R, Ray A, Ray P (1998). Essential role of nuclear factor kappaB in the induction of eosinophilia in allergic airway inflammation. J Exp Med..

[36] Zhang XY, Simpson JL, Powell H, Yang IA, Upham JW, Reynolds PN (2014). Full blood count parameters for the detection of asthma inflammatory phenotypes. Clin Exp Allergy..

[37] Hernandez-Pacheco N, Kere M, Melén E (2022). Gene-environment interactions in childhood asthma revisited; expanding the interaction concept. Pediatr Allergy Immunol..

[38] Güngör D, Nadaud P, LaPergola CC, Dreibelbis C, Wong YP, Terry N (2019). Infant milk-feeding practices and food allergies, allergic rhinitis, atopic dermatitis, and asthma throughout the life span: a systematic review. Am J Clin Nutr..

[39] Sheehan WJ, Phipatanakul W (2016). Indoor allergen exposure and asthma outcomes. Curr Opin Pediatr..

[40] Gold DR (2000). Environmental tobacco smoke, indoor allergens, and childhood asthma. Environ Health Perspect..

[41] Gergen PJ, Mitchell HE, Calatroni A, Sever ML, Cohn RD, Salo PM (2018). Sensitization and exposure to pets: the effect on asthma morbidity in the US population. J Allergy Clin Immunol Pract..

[42] Grant TL, Wood RA (2022). The influence of urban exposures and residence on childhood asthma. Pediatr Allergy Immunol..

